# Lokales Wirbelsäulenprofil nach operativer Behandlung thorakolumbaler und lumbaler Frakturen: Einfluss von Repositionstechnik und Knochenqualität

**DOI:** 10.1007/s00113-021-01013-7

**Published:** 2021-06-10

**Authors:** Bernhard Wilhelm Ullrich, Merle Ottich, Aaron Lawson McLean, Thomas Mendel, Gunther Olaf Hofmann, Philipp Schenk

**Affiliations:** 1 Klinik für Unfall‑, Hand- und Wiederherstellungschirurgie, Universitätsklinikum Jena, Am Klinikum 1, 07747 Jena, Deutschland; 2 Klinik für Unfall- und Wiederherstellungschirurgie, BG Klinikum Bergmannstrost Halle, Halle, Deutschland; 3 Klinik für Neurochirurgie, Universitätsklinikum Jena, Jena, Deutschland; 4 Koordinationsabteilung Wissenschaft, Forschung und Lehre, BG Klinikum Bergmannstrost Halle, Halle, Deutschland

**Keywords:** Hounsfield Unit, Repositionstechnik, Posttraumatische Kyphose, Repositionsverlust, Knochenqualität, Hounsfield Unit, Posttraumatic kyphosis, Reduction technique, Reduction loss, Bone quality

## Abstract

**Hintergrund:**

Ziel der Operation von Wirbelsäulenverletzungen ist eine stabile Ausheilung in physiologischer Stellung. Für offene und perkutane Operationen stehen unterschiedliche Techniken zur Verfügung.

**Fragestellung:**

Das Ausmaß der offenen Reposition und das Retentionspotenzial der Techniken nach AOSpine (AT) und nach Kluger (KT) sollen verglichen werden. Der Einfluss von Frakturmorphologie, Alter, Geschlecht und Knochenqualität auf Reposition und Retention werden untersucht.

**Material und Methoden:**

In dieser monozentrischen retrospektiven Kohortenstudie wurden Daten von Patienten mit traumatischen thorakolumbalen und lumbalen Frakturen untersucht, welche entweder mit AT oder KT reponiert wurden. Mittels bisegmentalen Grund-Deckplatten-Winkels (bGDW) wurde die Stellung des verletzten Wirbelsäulenabschnitts beschrieben. Normalwerte für die bGDW wurden anhand von Literaturdaten angenommen. Die Veränderung des bGDW im zeitlichen Verlauf wurde unter Einbeziehung der Knochenqualität in Hounsfield Units (HU), der Verletzungsschwere nach AOSpine und des Patientenalters und -geschlechts analysiert.

**Ergebnisse:**

Es wurden 151 Datensätze ausgewertet. Beide Methoden reponieren vom Umfang nicht unterschiedlich (AT 10 ± 6°, KT 11 ± 8°; *p* = 0,786). Im Follow-up trat ein Korrekturverlust von −5 ± 4° auf. Die Technik (*p* = 0,998) hatte keinen Einfluss darauf. Die Frakturmorphologie zeigte einen knapp signifikanten Einfluss (*p* = 0,043). Niedrige HU korrelierten mit geringerem Repositionsumfang (r = 0,241, *p* < 0,003) und größerem Korrekturverlust (r = 0,272, *p* < 0,001) signifikant, aber schwach. In der Altersgruppe 50 bis 65 Jahre wiesen 21 % der Männer und 43 % der Frauen eine Knochenqualität von HU < 110 auf. Alter und HU korrelieren signifikant (r = −0,701, *p* < 0,001).

**Diskussion:**

Die Techniken sind gleichwertig bezüglich der Repositions- und Retentionseigenschaften. Der hohe Anteil von Patienten mit HU < 110 in der Gruppe unter 65 Jahren bei Frauen und Männern und der Einfluss auf Reposition und Retention weisen auf die Notwendigkeit einer präoperativen Knochendichtemessung hin.

## Abstract (en)

**Background:**

The goal of surgery for spinal injuries is fracture reduction, fixation and stable healing in a physiological position. Several open and minimally invasive surgical techniques are available.

**Objective:**

The extent of open reduction and the fixation potential achieved by the AOSpine (AT) and Kluger (KT) techniques were compared. The influence of fracture morphology, age, sex, and bone quality on fracture reduction and secure fixation was investigated.

**Material and methods:**

In this monocentric retrospective cohort study data of patients with traumatic thoracolumbar and lumbar fractures treated by AT or KT were analyzed. The bisegmental kyphotic angle (bGDW) of each injured spinal segment was determined. Normal bGDW values were extrapolated from the literature. The change of bGDW over time was analyzed under consideration of the bone quality in Hounsfield units (HU), injury severity according to the AOSpine classification, gender and age of patients.

**Results:**

A total of 151 data sets were evaluated. The AT and KT methods achieved a similar extent of reduction (AT 10 ± 6°, KT 11 ± 8°; *p* = 0.786). In follow-up a mean reduction loss of −5 ± 4° was seen. The technique had no influence on this (*p* = 0.998). The fracture morphology just managed to achieve a significant influence (*p* = 0.043). Low HU correlated significantly but weakly with lower extent of reduction (r = 0.241, *p* < 0.003) and greater reduction loss (r = 0.272, *p* < 0.001). In the age group 50–65 years 21% of men and 43% of women had bone quality of < 110 HU. Age and HU were significantly correlated (r = −0.701, *p* < 0.001).

**Conclusion:**

The AT and KT are equivalent in terms of reduction and secure fixation properties. The high proportion of male and female patients with HU < 110 in the age group under 65 years and the influence on reduction and secure fixation emphasize the need for preoperative bone densitometry.

## Einleitung

Wirbelsäulenverletzungen haben, weltweit betrachtet, eine Inzidenz von 10,5/100.000. 48,8 % der Traumata werden operativ behandelt [1]. Minimal-invasive und offene Operationstechniken kommen dabei zur Anwendung.

Gegenstand dieser Studie ist der Vergleich der offenen Repositionstechniken nach AO Foundation (Davos, Switzerland) [2] und der von Kluger und Gerner beschriebenen [3]. Untersucht werden soll, ob beide Techniken hinsichtlich der lordosierenden Reposition gleichwertig sind. Anschließend soll ein möglicher Verlust der Reposition im zeitlichen Verlauf unter Berücksichtigung von Knochenqualität in Hounsfield Units (HU), der Frakturschwere nach AOSpine-Klassifikation [4] und des Patientenalters betrachtet werden.

## Methodik

Retrospektiv wurde das Krankenhausinformationssystem (ORBIS; Fa. Dedalus Healthcare Group, Florenz, Italien) eines überregionalen Traumazentrums nach chirurgisch behandelten Patienten mit singulären thorakolumbalen und lumbalen Wirbelsäulenverletzungen (Th10–L5) aus den Jahren 2001–2016 durchsucht. Eingeschlossen wurden Patienten, welche initial bisegmental dorsal offen instrumentiert wurden. Entsprechend dem im Erfassungszeitraum etablierten Therapiestandard erfolgte eine Verlaufsuntersuchung ca. 6 Wochen nach initialer dorsaler Stabilisierung vor ventraler Operation. Betrachtet werden nur die Daten vor ventraler Operation.

Im Bilddokumentationssystem IMPAX6® (Agfa HealthCare GmbH, Bonn, Germany) wurden die Datensätze dieser Patienten herausgesucht. Hierbei war Voraussetzung, dass es Röntgen- und CT-Aufnahmen präoperativ, direkt postoperativ und Verlaufsdaten gab.

Erfasst wurde neben Alter und Geschlecht die verwendete Operationstechnik (AO-Schanz-Pin-Technik [AT] und „Kluger Technik“ [KT]). In einem interdisziplinären Dienstsystem aus 3 Kliniken (neurochirurgisch, orthopädisch und unfallchirurgisch) waren Operateure beider Schulen (AT und KT) an der Akutversorgung von Wirbelsäulenverletzungen beteiligt. Alle Operateure waren Oberärzte und Fachärzte. Die Knochenqualität wurde mittels HU gemessen, die Frakturmorphologie nach AOSpine klassifiziert und der bisegmentale Grund-Deckplatten-Winkel (bGDW) auf Höhe der Verletzung gemessen [5].

**Figure 1:**
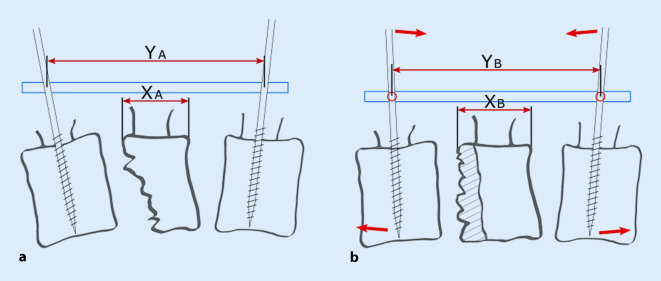

**Figure 2:**
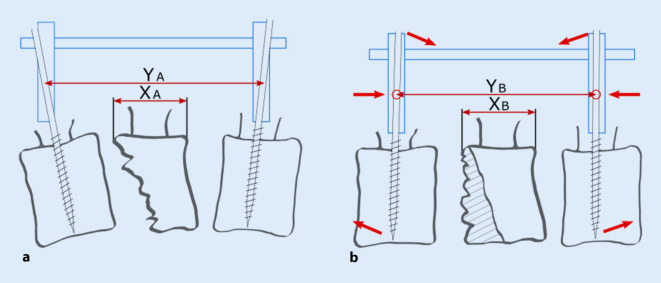

Die AO-Schanz-Pin-Technik steht seit Mitte der 1980er-Jahre zur Verfügung [6]. Diese nutzt den einliegenden Stab zur Reposition. Aktuell ist die Anwendung dieses Fixateur-Systems in den „surgical references“ der AOSpine beschrieben [2]. Hierbei wird nach Einbringen der Pins der kraniokaudale Abstand der Pins dorsal auf dem bereits eingebrachten Stab fixiert und dann eine Lordosierung und Distraktion durchgeführt (AT), Abb. 1. Ungefähr zeitgleich wurde die „Kluger-Technik“ beschrieben, welche mit einem temporär zu montierenden Repositeur arbeitet [3]. Mit diesem wird über Verlängerungen der Pedikelschrauben beim Lordosieren eine kontrollierte Verkürzung der kraniokaudalen Distanz der Pedikelschraubenköpfe durchgeführt, was eine stärkere Lordosierung ermöglichen soll (KT). Die Distraktion wird nach Fixation der lordotischen Stellung durchgeführt (krypton® [Ulrich, Ulm, Germany] Repositionstechnik WS 2801 Rev01/11-06), Abb. 2.

Die Methoden unterscheiden sich darin, dass bei der KT eine Annährung in den Facettengelenken während der Lordosierung möglich wird, welche bei der AT wegen der Längenfixation gegen den Stab vor der Lordosierung nicht möglich ist.

Die HU wurden in der Technik nach Schreiber erhoben [7]. Hierzu wurden die HU aus jeweils 3 Ellipsen in axialen Schichten (kranial, mittig und kaudal) eines an den frakturierten Wirbelkörper angrenzenden Wirbelkörpers gemittelt. Die Flächen der Ellipsen wurden maximal gewählt, ohne dabei kortikale Anteile zu enthalten. Ein Schwellwert von 110 HU wurde als Grenze zur Osteoporose entsprechend den Untersuchungen von Pickhardt et al. an 1867 CT- und DEXA-Datensätzen festgelegt [8].

Die Einteilung der Frakturschwere erfolgte anhand der AOSpine-Klassifikation [4]. Aufgrund der untersuchten lordosierenden Charakteristik beider Operationstechniken wurden Patienten mit B3-Frakturen in der Analyse nicht berücksichtigt, da hier i. d. R. eine kyphosierende Korrektur erforderlich ist.

Der bGDW wurde in Röntgen- und CT-Aufnahmen zu den Zeitpunkten präoperativ, postoperativ und zur Nachuntersuchung (einen bis 3 Monate) gemessen. Negative Werte zeigen eine kyphotische und positive Werte eine lordotische Stellung des bGDW an.

### Normwerte für bGDW

Auf der Grundlage der von Roussouly et al. [9] und Barrey et al. [10] erhobenen Werte für die lumbale Lordose (LL) und den „upper“ und „lower arc“ der LL wurden Normwerte für den bGDW abgeleitet, welche in Tab. 1 dargestellt sind. Für eine Beschreibung der traumatisch bedingten Fehlstellung wurde die Differenz zwischen gemessenem bGDW zum präoperativen Zeitpunkt mit diesen Werten gebildet.

**Table 1:** 

Bisegmentales Segment	Frakturierter Wirbel	Bisegmentale Normwerte
Th9/Th11	Th10	−5°
Th10/Th12	Th11	−2°
Th11/L1	Th12	2°
Th12/L2	L1	7°
L1/L3	L2	10°
L2/L4	L3	15°
L3/L5	L4	25°
L4/SWK 1	L5	40°

### Statistische Verfahren

Intervallskalierte Daten wurden mittels Kolmogorov-Smirnov-Tests auf Normalverteilung überprüft. Unterschiede zu separaten Zeitpunkten zwischen den beiden Operationstechniken wurden verteilungsadäquat mit dem Mann-Whitney-U-Test oder T‑Test für unabhängige Stichproben untersucht. Der Vergleich der bGDW mit den Normwerten erfolgte mittels verteilungsadäquaten Tests für abhängige Stichproben (*t*-Test oder Wilcoxon-Test) separat für beide Operationstechniken.

Mittels Chi-Quadrat-Test wurden folgende Inhalte geprüft: Verteilung der AOSpine in den Gruppen AT und KT, Unterschiede im Erreichen der Normwerte zwischen den Gruppen AT und KT sowie Unterschiede im Anteil von Patienten mit weniger als 110 HU in den Gruppen AT und KT.

Der Einfluss der Operationstechnik auf die Änderung des bGDW wurde mithilfe separater allgemeiner lineare Modelle für Messwiederholungen (rmANCOVA (Zeitpunkte (2) × Operationstechnik (2))) untersucht. Aufgrund des *p*-Wertes und eines möglichen Einflusses der AOSpine-Klassifikationen auf das Repositionsergebnis zwischen in den Operationsgruppen (AT und KT) wurde diese als Kovariate verwendet.

Der Einfluss der HU auf den Umfang der Reposition und auf den Umfang der Änderung des bGDW im postoperativen Verlauf wurde mittels Pearson-Korrelation untersucht. Die Spearman-Korrelation wurde verwendet, um den Zusammenhang zwischen Frakturmorphologie nach AOSpine und dem Umfang der Reposition und der postoperativen Änderung des bGDW zu untersuchen.

Der Zusammenhang zwischen Alter und Knochenqualität wurde mittels Pearson-Korrelation untersucht. Unterschiede in der Knochenqualität zwischen den Geschlechtern wurde zum einen mittels *t*-Test für unabhängige Stichproben untersucht und zum anderen altersadjustiert mittels einer univariaten Varianzanalyse.

Für die statistischen Analysen wurde SPSS V26 (IBM Corp. IBM SPSS Statistics for Windows; Fa. IBM Corp, Armonk, NY) verwendet.

## Ergebnisse

Es wurden Daten von 1538 Patienten gesichtet. Insgesamt konnten vollständige Datensätze von 151 Patienten (109 Männer, 42 Frauen) analysiert werden. Das Alter der Patienten lag im Mittel bei 45 ± 16 Jahren und unterschied sich nicht zwischen beiden Operationsgruppen (*p* = 0,415). Das Durchschnittsalter der Frauen (48 ± 16 Jahre) lag im Mittel nur 3 Jahre über dem der Männer (45 ± 16 Jahre, *p* = 0,328). Der Zeitraum der Nachuntersuchung betrug im Median 50 Tage (*p* = 0,069). Entsprechend der Operationstechnik wurden 102 Patienten der Gruppe AT (77 %) und 49 der Gruppe KT (23 %) zugeordnet. Die Verletzungshöhen und deren Verteilung auf die Gruppen AT und KT sind in Tab. 2 dargestellt. Unterschiede in der Verteilung der Häufigkeit der frakturierten Wirbel zwischen beiden Gruppen bestanden nicht (*p* = 0,465). Frakturen von Th12, L1 und L2 machten 82 % aller Verletzungen aus.

**Table 2:** 

		AT, *n* (%)	KT, *n* (%)	Gesamt, *n* (%)
Wirbel	Th10	1 (1)	1 (2)	2 (1)
	Th11	3 (3)	1 (2)	4 (3)
	Th12	20 (20)	13 (27)	33 (22)
	L1	45 (44)	22 (45)	67 (44)
	L2	14 (14)	7 (14)	21 (14)
	L3	9 (9)	–	9 (6)
	L4	7 (7)	4 (6)	10 (7)
	L5	3 (3)	2 (4)	5 (3)
Gesamt		102	49	151

Die Häufigkeiten der Frakturmorphologie nach AOSpine-Klassifikation sind in Tab. 3 dargestellt. Es waren Frakturen von A1–C vertreten; Patienten mit B3 Frakturen wurden ausgeschlossen. A4-Frakturen traten in beiden Gruppen am häufigsten auf (AT: 49 %, KT: 74 %). Mit 22 % in der Gruppe AT und 17 % der Gruppe KT waren A3-Frakturen am zweithäufigsten. In Summe verteilen sich 74 % der Frakturen auf diese beiden Frakturtypen. Im Mittel zeigten die Patienten der Gruppe AT knapp keine unterschiedliche Verteilung der Frakturtypen nach AOSpine als die der KT-Gruppe (*p* = 0,058).

**Table 3:** 

		AT, *n* (%)	KT, *n* (%)	Gesamt, *n* (%)
AOSpine	A1	4 (4)	–	4 (3)
	A2	7 (7)	1 (2)	8 (5)
	A3	22 (22)	4 (8)	26 (17)
	A4	50 (49)	36 (74)	86 (57)
	B1	9 (9)	3 (6)	12 (8)
	B2	3 (3)	2 (4)	5 (3)
	C	7 (7)	3 (6)	10 (7)
Gesamt		102	49	151

### Frakturbedingte Deformität

**Figure 3:**
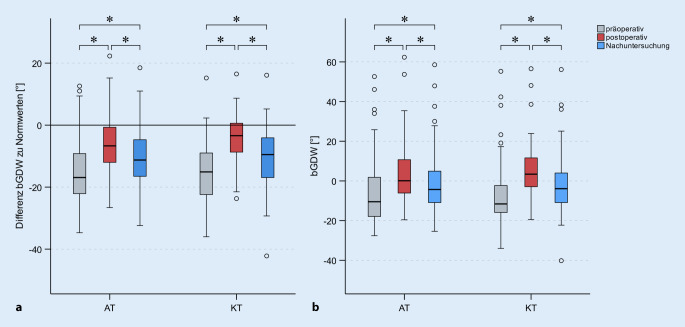

Verglichen mit den Normwerten zeigte sich präoperativ in beiden Gruppen eine signifikante frakturbedingte kyphotische Fehlstellung (AT: −15 ± 10°, *p* < 0,001; KT: −15 ± 10°, *p* < 0,001), welche sich zwischen den Operationsgruppen nicht sign. unterschied (*p* = 0,837, Abb. 3a). Die Nulllinie stellt hierbei den angenommenen Normwert höhenabhängig als Referenz dar. Die gemessenen präoperativen bGDW der Patienten unterschieden sich ebenfalls nicht zwischen beiden Operationsverfahren (AT: −6 ± 16°, KT: −6 ± 18°, *p* = 0,798, Abb. 3b).

### Repositionsergebnis

Durch die Operation wird eine Änderung der bGDW in der Gesamtpopulation von 11 ± 8 (*p* < 0,001) herbeigeführt. Mit der AT wurde eine Reposition um 10 ± 6 und mit der KT um 11 ± 8 erzielt. Beide Operationstechniken unterscheiden sich nicht signifikant im Umfang der erzielten Reposition (*p* = 0,786). Der Interaktionseffekt zwischen Zeitpunkt und Operationstechnik war ebenfalls nicht signifikant (*p* = 0,476). Die Frakturschwere nach AOSpine als Kovariate zeigt keinen signifikanten Einfluss (*p* = 0,225) auf den Umfang der Reposition.

Bei 40 (26 %) Patienten (AT: 24 %, KT: 33 %, *p* = 0,244) wurden die Normwerte der bGDW durch die Reposition erreicht. Bei allen anderen Patienten (76 %) verblieb postoperativ ein kyphotischer Fehlstellungswinkel im Vergleich zu den Normwerten. Im Mittel unterschieden sich die postoperativen bGDW beider Gruppen signifikant von den Normwerten (AT −5 ± 8°, *p* < 0,001; KT −4 ± 8°, *p* < 0,001).

### Postoperativer Verlauf

Der bGDW verringerte sich im postoperativen Verlauf um −5 ± 4 signifikant (*p* < 0,001). Die Operationstechnik (*p* = 0,998) zeigt sich nicht signifikant, die Verletzungsschwere (*p* = 0,043) hingegen zeigt einen knapp signifikanten Einfluss. Zur Nachuntersuchung betrugen die mittleren bGDW für AT = −1 ± 15° und für KT = −1 ± 16° (*p* = 0,930). Verglichen mit den präoperativen bGDW verbleibt ein mittleres Repositionsergebnis von 5 ± 8 (*p* = 0,002). Beide Operationstechniken unterscheiden sich nicht im verbliebenen Repositionsergebnis (*p* = 0,290). Ein Zusammenhang zwischen Umfang der ursprünglichen Reposition und dem Umfang des Repositionsverlusts lässt sich für beide Operationstechniken (AT *p* = 0,912, KT *p* = 0,420) und über die komplette Stichprobe (*p* = 0,430) nicht zeigen.

Zum Zeitpunkt der Nachuntersuchung weisen nur noch 22 (16 %) Patienten (AT: 16 %, KT: 12 %, *p* = 0,632) einen bGDW oberhalb der Normwerte auf.

### Hounsfield Units

**Figure 4:**
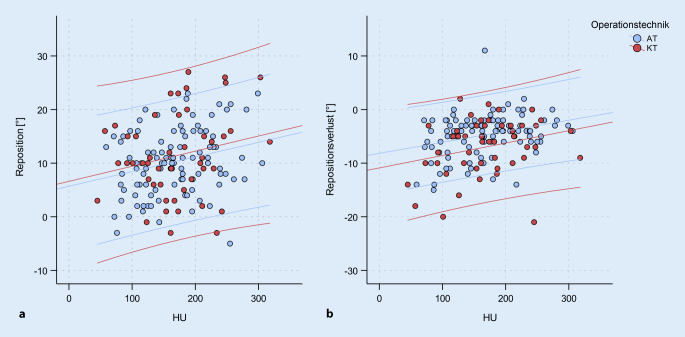

Die HU-Werte lagen zwischen 45 und 318 (168 ± 58 HU). Unterschiede zwischen beiden Operationsgruppen konnten nicht festgestellt werden (*p* = 0,714). Bei insgesamt 28 Patienten (19 %) wurden HU-Werte kleiner als 110 gemessen. Der Anteil an Patienten mit HU kleiner als 110 war in beiden Operationsgruppen vergleichbar (AT: 20 %, KT: 16 %, *p* = 0,823). Der Zusammenhang zwischen der Knochenqualität und dem Umfang der Reposition ist signifikant (r = 0,241, *p* = 0,003). Dies bedeutet, dass bei Patienten mit höheren HU eine umfangreichere Reposition erzielt wurde. Mit einem r^2^ von 0,06 ist dieser Zusammenhang jedoch als sehr gering zu bezeichnen (Abb. 4a). Auf den Repositionsverlust im Nachuntersuchungszeitraum zeigen die HU-Werte ebenfalls einen signifikanten Einfluss (r = 0,272, *p* = 0,001). Niedrigere HU sind demnach mit einem größeren Repositionsverlust verbunden (Abb. 4b). Auch hier ist der Zusammenhang als gering zu bezeichnen (r^2^ = 0,07).

### AOSpine-Klassifikation

**Figure 5:**
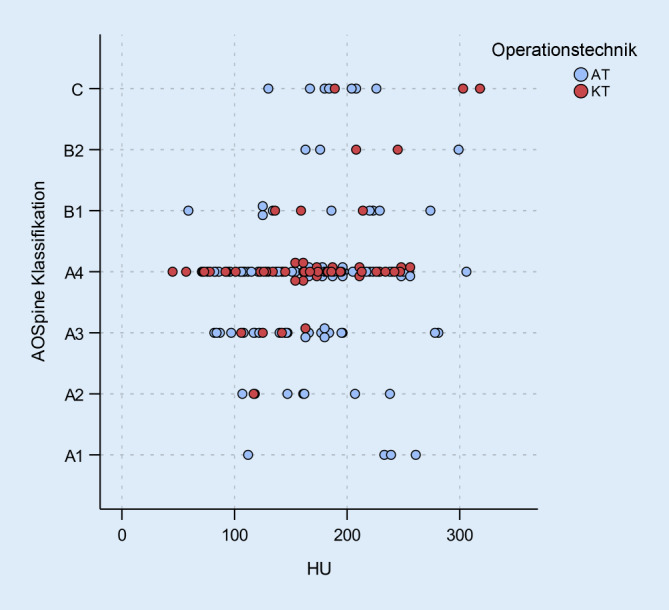

Ein Zusammenhang zwischen der Frakturmorphologie und der erzielten Reposition besteht schwach, aber signifikant (r = 0,189, *p* = 0,020) in dem Sinne, dass bei einer höhergradigen Fraktur auch ein größerer Repositionsumfang erzielt wird. Für den Korrekturverlust findet sich dieser Zusammenhang nicht (*p* = 0,457). Der Zusammenhang zwischen Knochenqualität und Frakturmorphologie zeigt sich als sehr schwach (r = 0,186), aber signifikant (*p* = 0,022). Patienten mit hohen HU-Werten zeigen eine Tendenz zu höhergradigeren Verletzungen nach AOSpine (Abb. 5).

### Beziehung HU, Alter, Geschlecht

Insgesamt 132 Patienten (87 %) waren jünger als 65 Jahre. Hiervon wiesen 17 (13 %) eine schlechte Knochenqualität (HU < 110) auf. Männer (*n* = 11 von 96) und Frauen (*n* = 6 von 36) verteilten sich somit zu 11 und 17 %.

Insgesamt 43 aller Patienten waren zwischen 50 und 65 Jahre alt. Hiervon wiesen 12 (43 %) (6 von 29 Männern (21 %) und 6 von 14 Frauen (43 %)) eine schlechte Knochenqualität (HU < 110) auf.

**Figure 6:**
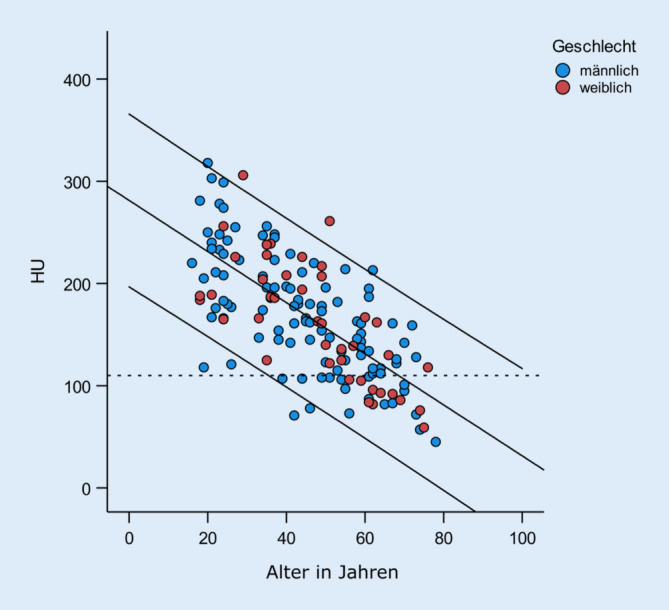

Mit zunehmendem Alter zeigt sich eine Verminderung der Knochenqualität (r = −0,701, *p* < 0,001). Der Zusammenhang ist als stark zu bezeichnen und in Abb. 6 dargestellt. Die geschlechtsspezifische Betrachtung der Knochenqualität zeigt im Mittel keine Unterschiede zwischen Männern (170 ± 58 HU) und Frauen (163 ± 59 HU, *p* = 0,518). Unter Berücksichtigung des Alters als signifikante Kovariate (*p* < 0,001) zeigt sich ebenfalls kein signifikanter Unterschied (*p* = 0,937). Die altersadjustierten Mittelwerte der HU über die komplette Stichprobe liegen für Männern bei 168 (95. Konfidenzintervall 160–176) und für Frauen bei 168 (95. Konfidenzintervall 156–181).

## Diskussion

Auffällig ist, dass nur ca. 10 % der durch die Suchanfrage ausgegebenen Datensätze für die Auswertung herangezogen werden konnten. Die häufigsten Gründe für die Reduktion bei gleichzeitiger Homogenisierung der Stichprobe waren: fehlende Verlaufsdokumentationen, unvollständige Röntgendokumentation, multisegmentale Stabilisierung, polyaxiale oder minimal-invasive Fixateur-Systeme. Da keine systematische Selektion stattgefunden hat, ist davon auszugehen, dass die Ergebnisse trotzdem repräsentativ sind. Primär engere Auswahlkriterien hätten eine präzisere Ausgabe ermöglicht. Die Übertragbarkeit und Generalisierung unserer Ergebnisse auf die ursprüngliche Kohorte ist somit aufgrund des geschrumpften Stichprobenumfangs eingeschränkt.

### Repositionstechniken

Durch die offene Reposition ist eine relevante Korrektur der unfallbedingten Fehlstellung möglich, wie auch in der zweiten internetbasierten Multizenterstudie der Deutschen Gesellschaft für Unfallchirurgie (MCS ll) gezeigt werden konnte [11]. Das Repositionsausmaß von 10 können wir bestätigen. Die Daten der MSC ll zeigen, dass das initiale Repositionsergebnis insbesondere ohne zusätzliche ventrale Stabilisierung nicht gehalten werden konnte [12]. Dass ein erheblicher Repositionsverlust im Zeitraum vor einer ventralen Stabilisierung eintritt, können wir mit dieser Untersuchung bestätigen. Daten aus der hier dargestellten Kohorte nach ventraler Abstützung durch einen „Stand-alone“-Wirbelkörperersatz (VLIFT® Stryker, Kalamazoo, MI, USA) zeigten aber auch, dass die Sinterung nach einer ventralen Stabilisierung von der Knochenqualität abhängig ist. Je schlechter die Knochenqualität, desto größer die Sinterung. Ausschließlich in der Gruppe mit HU-Werten > 180 konnte eine minimale Sinterung detektiert werden [13].

### Korrekturverlust

Der Korrekturverlust von im Mittel −5 bei alleiniger dorsaler Instrumentierung ist signifikant und stützt die Ergebnisse der MSC ll (−7°) und der Arbeit von Spiegl et al. (−5 nach 60 Tagen) [14]. Aussagen zur klinischen Bedeutung dieser Fehlstellung können nicht getroffen werden, da im Rahmen dieser Untersuchung keine strukturierte Erfassung klinischer Parameter erfolgte. In einer monozentrischen prospektiven Pilotstudie, bei der die Indikation zur anterioren Fusion randomisiert gestellt wurde, bestätigte sich ein signifikant geringerer Repositionsverlust und zeigten sich tendenziell klinisch bessere Ergebnisse im ODI für die dorsoventral stabilisierten Patienten [15]. Der Wert der additiven ventralen Stabilisierung wird weiterhin kritisch diskutiert, wie in einem Review von Tan et al. aus 2020 zum Ausdruck kommt, welches allerdings die MSC-Daten [11, 12, 16] nicht berücksichtigt [17].

### Knochenqualität

Die Knochenqualität wurde in der MSC ll nicht betrachtet. HU aus opportunistischen CT-Scans können als Surrogatparameter für die Knochenqualität herangezogen werden [7, 8]. Pickhardt et al. [8] belegten, dass Werte < 110 HU eine mehr als 90 %ige Spezifität für die Vorhersage einer Osteoporose haben. Legt man dies zugrunde, zeigen die hier vorgestellten Daten, dass ein relevanter Anteil der unter 65-jährigen Patienten (Frauen 17 %, Männer 11 %) sehr wahrscheinlich eine osteoporotische Knochenqualität aufwies. Unabhängig davon zeigen niedrige HU-Werte ein erhöhtes Risiko für Komplikationen wie Sinterungen von Cages [13], Lockerungen von Pedikelschrauben [18] und Anschlussfrakturen [19] an. Sie können somit dienlich für die Operationsplanung sein. Die hier erhobenen Daten zeigen einen signifikanten Einfluss der HU auf das Repositionsergebnis (je niedriger die HU, umso kleiner der Repositionsweg) und den Korrekturverlust (je niedriger die HU, umso größer der Repositionsverlust). Aufgrund der breiten Streuung der Werte sind die gefundenen Zusammenhänge als sehr gering zu bezeichnen, und die Aussagekraft der Ergebnisse ist limitiert.

Interessant an den Daten der MSC ll ist, dass ab einem Patientenalter von 41 banale Stürze einen relevanten Anteil der Unfallursache darstellen. Dies deutet darauf hin, dass der Anteil an Patienten mit verminderter Knochenqualität in der MCS ll auch bei Patienten zwischen 40 und 60 Jahren höher gewesen sein könnte als angenommen.

### Frakturbedingte Deformität

Das Ziel der Reposition ist die Korrektur der Fehlstellung auf das ursprüngliche, individuelle Wirbelsäulenprofil. In der Regel existieren keine prätraumatischen Aufnahmen der Wirbelsäule. Es kommt hinzu, dass bei wirbelsäulenverletzten Patienten Untersuchungen der Wirbelsäule gemäß der „Leitlinie der Bundesärztekammer zur Qualitätssicherung in der Röntgendiagnostik“ [20] im Stehen, die üblicherweise zur Feststellung des individuellen sagittalen und koronaren Profils zur Anwendung kommen, nicht möglich sind. Wirbelsäulenverletzungen bedingen in der Regel eine kyphotische Deformität, die im Verlauf erheblich zunehmen kann [21], sodass eine Bestimmung des individuellen Profils, wie es vor der Verletzung bestanden hat, nicht möglich ist.

### Prätraumatisches lokales Wirbelsäulenprofil

Nach Kenntnis der Autoren gibt es kein etabliertes Verfahren, mit welchem bei einem im Liegen untersuchten wirbelsäulenverletzten Patienten vorbestehende physiologische bGDW der verletzten Segmente abgeleitet werden können. Um dieses Problem zu adressieren, wurden für diese Arbeit, basierend auf den Daten von Roussouly et al. [9] und Barrey et al. [10], Normwerte angenommen. Kritisch daran ist, dass die starken interindividuellen Schwankungen mit diesen Mittelwerten nicht erfasst werden. Betrachtet man die Lordosetypen nach LeHuec und die damit einhergehende Varianz der Höhe des Inflexionspunktes [9] kann der physiologische Wert für den bGDW der Segmente Th12/L1/L2 zwischen −10 und +10° variieren.

Dass durch die Reposition die angenommenen Normwerte nur in einer Minderzahl der Fälle erreicht wurden, kann durch oben genannte Limitation bedingt sein oder tatsächlich durch einen unzureichenden Repositionsweg verursacht sein. Diese Frage kann mit den vorhandenen Daten nicht geprüft werden.

Seit 2013 steht als Ergebnis eines globalen Konsensusprozesses die AOSpine-Klassifikation für die Einteilung der Schwere thorakolumbaler Verletzungen zur Verfügung [4], welche vorangegangene Klassifikationen wie die nach Magerl [22], McCormack [23] und die TLICS [24] aufgenommen und zusammengeführt hat. Somit scheint die AOSpine-Klassifikation die Verletzungsschwere umfänglich zu beschreiben, sodass auf eine Darstellung der Ergebnisse im Bezug zu den anderen Klassifikationen (Magerl, McCormack und TLICS) nicht erfolgte.

Dass bei Patienten mit niedrigeren HU eher niedriggradigere Frakturen auftreten, kann mit den vorliegenden Daten nicht erklärt werden. Möglicherweise führen Niedrigenergietraumen bei Patienten mit schlechterer Knochenqualität häufiger zu einfacheren Frakturen als bei Knochengesunden. Informationen zum Unfallhergang mit einer Kategorisierung nach einwirkender Energie in Analogie zur MSC ll wären geeignet, den Unfallhergang als Kovariate zu untersuchen.

### HU, Alter und Geschlecht

Die Ergebnisse zeigen die bekannte negative Korrelation [25] zwischen Alter und HU. Interessant ist, dass sich altersbereinigt kein Unterschied zwischen Männern und Frauen finden lässt. Demnach scheint in unserer Kohorte das Alter einen stärkeren Einfluss auf die HU zu haben als das Geschlecht. Mögliche Ursache hierfür kann sein, dass das Vorhandensein einer Wirbelfraktur im Zusammenhang mit der Knochenqualität steht und somit dieses Ergebnis auf einem Selektionsbias beruht.

## Fazit für die Praxis

- Die AO-Schanz-Pin-Technik und die Kluger-Technik sind gleichwertig geeignet, thorakolumbale und lumbale Wirbelfrakturen zu reponieren.
- Der Anteil von Patienten mit erheblich reduzierter Knochenqualität liegt bei unter 65-Jährigen in einem für die Therapie relevanten Ausmaß.
- Die Hounsfield Unit-Werte aus nativen CT-Scans als Maß für die Knochenqualität haben einen Einfluss auf das Repositionsergebnis und den Korrekturverlust und sollten präoperativ bestimmt werden.
- Bereits nach kurzer Zeit ist unabhängig von der Operationstechnik ein signifikanter Repositionsverlust nachweisbar.
